# The Practice of Surgery: Embracing Minor Surgery, and the Application of Dressings, Etc., Etc.

**Published:** 1849-12

**Authors:** 


Art. VII.—*The Practice of Surgery: embracing Minor Surgery and the Application of Dressings, etc., etc.* By John Hastings, M. D., U. S. N., Fellow of the College of Physicians of Philadelphia, Lecturer on Surgical Anatomy and Operative Surgery, etc., etc. With numerous illustrations. Philadelphia: Lindsay & Blackiston. 1850.

We have examined this work with some degree of care, and though it is not, by any means, all that it should be, we commend it to the attention of the general practitioner. In all compilations of this kind there must be serious defects, from the fact that the compiler is apt to follow authority, rather than observation, and he often runs after an ignis fatuus, when he feels persuaded that he is guided by truth. A familiar illustration of this remark may be found in the subject of Hydrocele. Dr. Hastings, in following the usual authorities, recommends the use of the trocar, and an injection of some stimulant articles, and he refers to the use of the seton. Neither of these plans is the best, for they often, and within our experience always, fail in curing. Professor Dudley has been in the habit, for many years, of making an extended incision entirely through the tunica vaginalis, and healing the wound from the bottom. This plan offers many advantages over the seton or the puncture. It is equally as safe to the patient as either of the others; it is much more successful than they are, and in addition to these recommendations, this method enables the surgeon to remove all doubts as to whether the case in hand is hernial, or encysted hydrocele, hydrocele of the tunica vaginalis, or hydrocele of the cord. It is not always possible to make the diagnosis perfectly in practice. Prof. Gross has recommended a plan, too, in a recent number of the American Journal of the Medical Sciences, which embraces the advantages we have mentioned, and Dr. Hastings does not refer in his work to either of the American operations. The practitioner, therefore, who may take Dr. Hastings as his guide, would not follow the best method of managing hydrocele.

But we cannot enter into a detail of the merits or demerits of the work. Dr. Hastings deserves credit for the industrious manner in which he has collated the authorities in surgery, and though he has not always, in our judgment, selected the best methods, he has made a very respectable vade mecum of surgical science and art.—There are several works of the kind before the profession, and this is probably equal to any of them.

The work before us is well printed, and has numerous illustrations of the various matters presented. We commend its perusal to young practitioners, and they will soon learn how far it may be followed as a guide.

This work, as well as Stanley on the "Diseases of the Bones," may be found at the bookstore of Beckwith & Morton.

